# A comparison of angled (D-Blade) and Macintosh (C-MAC) videolaryngoscopes for simulated pediatric difficult airway: a randomized single-blind study

**DOI:** 10.3906/sag-2102-221

**Published:** 2021-11-27

**Authors:** Alparslan KUŞ, Can AKSU, Hadi Ufuk YÖRÜKOĞLU, Volkan ALPARSLAN, Kamil TOKER

**Affiliations:** 1 Department of Anesthesiology and Reanimation, Faculty of Medicine, Kocaeli University, Kocaeli, Turkey; 2 Clinic of Anesthesiology and Reanimation, Tatvan State Hospital, Bitlis, Turkey; 3 Clinic of Anesthesiology and Reanimation, Hatay State Hospital, Hatay, Turkey; 4 Department of Anesthesiology and Reanimation, Faculty of Medicine, İstinye University, İstanbul, Turkey

**Keywords:** Pediatric airway, difficult airway, videolaryngoscope

## Abstract

**Background/aim:**

Being prepared for difficult airway (DA) is nevertheless of great importance. Failed or delayed tracheal intubation (TI) can increase morbidity and mortality, and the pediatric population is more prone to hypoxia. With the development of different types of videolaryngoscope (VL), these have become the device of choice in patients with DA.

Our primary aim was to compare intubation times with D-blade and Macintosh blade of Storz C-MAC^®^ in a simulated pediatric DA scenario with this randomized controlled trial.

**Materials and methods:**

Children aged 1–5 years scheduled for elective surgery were included in the study. Patients were randomized into two groups: the D-Blade (n = 20) and MAC (n = 21) groups. All children underwent inhalational induction, and a neuromuscular relaxant was routinely administered (rocuronium 0.6 mg.kg-1). After the appropriate size of semirigid foam neck collar had been positioned around the patient’s neck, the D-Blade group patients were intubated using a size 2 D-Blade, and the MAC group patients used a size 2 VL Macintosh blade. Intubation, time was measured. Patients’ modified Cormack-Lehane system scores (MCLS), pre and postintubation blood pressure values and heart rates, and complications during intubation were recorded.

**Results:**

Demographic data were similar between the groups. There were also no significant differences in pre and postintubation heart rates, blood pressure, or SpO2 values (p > 0.05 for all). Mean intubation times for the MAC and D-Blade groups were 12.14 ± 2.79 s and 18.31 ± 10.86 s, respectively (p = 0.022). MCLS scores were lower in the D-Blade group (p = 0.030).

Intubation success rates were 100% in the MAC group and 90% in the D-Blade group, although the difference was statistically insignificant (p = 0.165).

**Conclusion:**

A better laryngoscopic view was obtained with D-Blade. However, the Storz® C-Mac videolaryngoscope Macintosh blade was superior to the D-Blade in achieving a shorter time for TI.

## 1. Introduction

The different anatomy and physiology of children distinguish pediatric airway management from that of adults. These differences lead clinicians to encounter more difficult airway (DA) events than in adults [1, 2].

Tracheal intubation (TI) represents the main element of pediatric airway management for safety. Although clinicians usually accomplish TI without difficulty with conventional direct laryngoscopy, being prepared for DA is nevertheless of great importance. Failed or delayed TI can increase morbidity and mortality because the pediatric population is more prone to hypoxia [1].

With the development of different types of videolaryngoscope (VL), these have become the device of choice in patients with DA [3, 4]. Awake TI with VLs has even become the subject of discussion in adult patients with anticipated DA [5, 6]. New types of blade have been developed due to this growing interest. The C-MAC® D-Blade (Karl Storz, Tuttlingen, Germany) is one of these clinically newly adopted blades. This blade is hyper-angulated with a half-moon shape, making it more compatible with the oropharynx’s anatomy [7]. Although the current literature supports this device’s superiority in DA scenarios in adult patients, data for the pediatric population are still insufficient [8].

**Figure 1:**
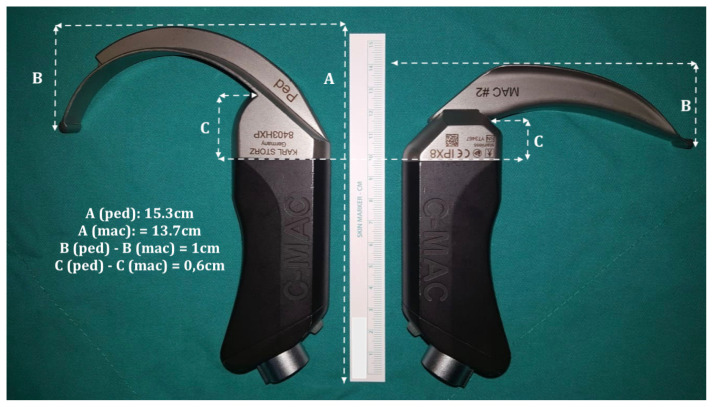
Visual comparison of the two blades. Differences in the design, angulation, and length of the blades are shown.

We hypothesized that due to its more massive structure, the C-MAC® D-Blade would occupy more space in the oral cavity than the standard C-MAC® blade, thus making endotracheal insertion and manipulation more difficult in pediatric patients (Figure 1). This randomized study aimed to compare these two VL blades in a simulated pediatric DA scenario. The primary end-point was to compare intubation times. The secondary end-point was to compare laryngoscopic views, complication rates, and success rates at the first attempt between the groups.

## 2. Materials and methods

This prospective, randomized controlled trial was performed after obtaining the Kocaeli University Ethical Committee of Clinical Research approval (KIA 2018-154) and written informed consent from the patients’ parents. The study was registered with clinicaltrials.gov (NCT03719638) and was conducted between November 2018 and February 2019.

Children aged 1–5 years, with American Society of Anesthesia (ASA) physical status I–II, and scheduled for elective surgery by the pediatric surgery department, were included in the study. Exclusion criteria were the presence of pulmonary or neuromuscular diseases, risk of pulmonary aspiration, presence of any pathology of the neck and head and anticipated DA.

Patients were randomized into two groups using computer-generated random number tables, a D-Blade group, and a MAC group. The D-Blade group patients were intubated using a size 2 Storz® C-Mac VL D-Blade, and MAC group patients using a size 2 Storz® C-Mac VL Macintosh blade by two same anesthesiology specialists (CA and HUY), who were both experienced in pediatric Storz® C-Mac videolaryngoscopy.

All children were monitored using electrocardiograms, noninvasive arterial blood pressure measurements, pulse oximetry, capnography, and inspired oxygen concentration measurements in the operating room.

All children underwent inhalational induction with 8% sevoflurane in a mixture of 66% nitrous oxide and 33% oxygen. A neuromuscular relaxant was routinely administered (rocuronium 0.6 mg.kg-1). The anesthesiologist waited for at least 3 min after rocuronium administration to ensure adequate laryngeal relaxation. All intubations were performed using an uncuffed endotracheal tube, shaped with a stylet related to the blade used, after the appropriate size of semirigid foam neck collar (Philadelphia cervical collar) had been positioned around the patient’s neck according to the manufacturer’s recommendations. Each laryngoscopy was scored using the modified Cormack-Lehane System (MCLS) [9]. This 5-grade scoring system involves the subdivision of the original grade 2 into 2A (partial view of glottis visible) and 2B (only the arytenoids visible). Intubation time was measured from raising the laryngoscope to the mouth above the teeth until confirmation of intubation, visualization of end tidal carbon dioxide (ETCO2), and chest expansion. Patients’ pre and postintubation blood pressure values, heart rates, and complications such as lip, dental, or mucosal injury (blood detected on the airway device) during intubation were recorded.

Patients were excluded if intubation was unsuccessful at the first attempt or peripheral oxygen saturation (SpO2) rates decreased below 92%. In these cases, the cervical collar was removed, and airway management was performed according to departmental guidelines.

### 2.1. Statistical analyses

A preliminary study in our clinic involving 10 patients revealed a mean (± SD) intubation time of 16.31 s (±6.47) using the Storz® C-Mac videolaryngoscope Macintosh blade. We calculated that for 80% power and an error of 0.05, the sample size necessary to detect a 25% difference in intubation time with the D-Blade compared to the MAC group would be 18 subjects for each group. Twenty-two patients were enrolled in each group against the possibility of dropouts.

All statistical analyses were performed on IBM SPSS for Windows® version 20.0 software (SPSS, Chicago, IL, USA). The Kolmogorov-Smirnov test was used to determine the normality of data distribution. Continuous variables were expressed as mean ± standard deviation, and categorical variables as counts (percentages). Comparisons of normally distributed continuous variables between the groups were performed using student’s t-test, while nonnormally distributed continuous variables between the groups were compared using the Mann Whitney U test. Comparisons of categorical variables between the groups were performed using the chi-Square test. A two-sided p-value < 0.05 was considered statistically significant.

## 3. Results

**Figure 2:**
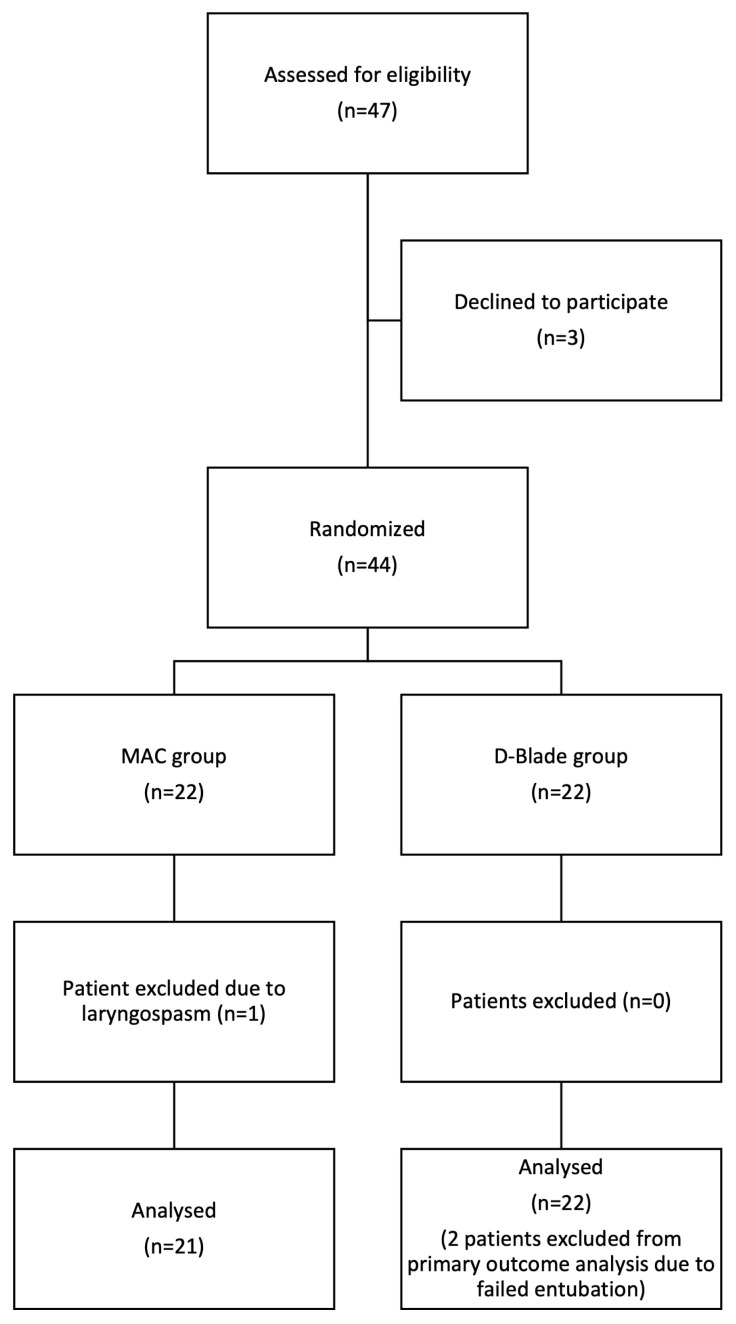
Consort flow diagram.

Forty-four parents consented to participate in the study. One patient from the MAC group was excluded due to laryngospasm during induction of anesthesia. Two patients in the D-Blade group were excluded from intubation time analysis due to failed intubation at the first attempt (Figure 2).

**Figure 3:**
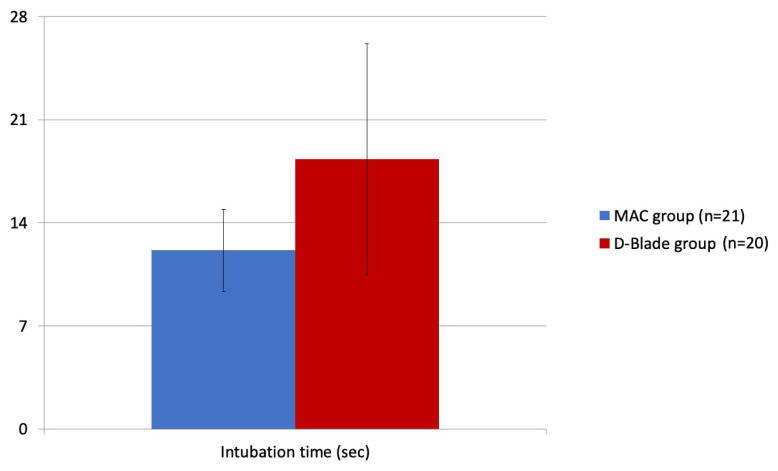
Intubation times. *p = 0.022

Demographic data were similar between the groups (Table 1). Preintubation heart rates were 112.4 ± 5.8 and 115.2 ± 4.9 beats per minute (bpm) and postintubation heart rates were 116.1 ± 6.2 and 116.5 ± 6.3 bpm, respectively for the MAC and D-Blade groups. There were no significant differences in pre and postintubation heart rates, or SpO2 values (p > 0.05). Mean intubation times for the MAC and D-Blade groups were 12.14 ± 2.79 s and 18.31 ± 10.86 s, respectively, the difference being statistically significant (p = 0.022) (Figure 3). Glottic visualization was better in the D-Blade group (p = 0.030) (Table 2).

Intubation success rates were 100% in the MAC group and 91% in the D-Blade group, although the difference was statistically insignificant (p = 0.165).

No mucosal, dental, or lip injuries were present in either group, and no study-related complications occurred.

## 4. Discussion

Our study results showed the Storz® C-Mac VL Macintosh blade shortened intubation times in pediatric patients with simulated DA, while the D-Blade provided a better laryngoscopic view in association with lower MCLS scores.

Time to intubation is as vital as successful intubation in pediatric airway management. Intubation times in the two groups differed by nearly 6.5 s. This difference may be clinically unimportant in adult patients, but in pediatric patients, especially in DA scenarios, every second may be crucial. A 5-s delay in TI has been shown to be potentially clinically significant in the pediatric population [10]. Limited mouth opening and oropharyngeal space hindered the insertion of the Storz D-Blade, which has a bulky structure with a highly angulated body, into the oral cavity in our study. This may account for the prolonged intubation time and two failed attempts. In addition to the D-Blade’s bulky nature, hyperangulation also makes it challenging to manipulate tube insertion, and this may be another reason for the prolongation.

Neck collars are generally used to simulate DA scenarios. In our study, the application of a neck collar reduced both head and neck movement and mouth opening. Reduced mouth opening has been reported in patients with neck collars [11]. Reduced neck movement leads to reduced head flexion, which contributes to the worsening of Cormack-Lehane grades with direct laryngoscopy [12].

In our study, as no patients had had higher MCLS scores than 2A, we identified the D-Blade as superior to the Mac Blade in terms of achieving better laryngoscopic views. We think that this may be due to the D-Blade’s hyperangulation, as also described in other studies. Likewise, Jain et al. [13] also found a better glottic view with the D-Blade than the Mac blade of C-MAC in an adult manikin study. However, manikin studies have some disadvantages and cannot be generalized to the average population.

Insertion of D-Blade was more difficult than MAC blade in our study, and it resulted in longer intubation times in the D-Blade group, which was statistically significant. We think that this difference was because of the hyperangulated structure of the D-Blade in addition to the bulky handle. It was reported that the relatively bulky handle of the size 2 MAC blade of C-MAC abutted the patient’s chest and prevented the full insertion of the blade [14].

Due to the hyperangulated structure of the D-Blade, a stylet must be used for tube insertion. We encountered two difficulties when a stylet matching the shape of the D-Blade was used. The first was the insertion of the tube from the oral cavity to the glottis area without seeing it until it appeared on-screen. This part of the insertion procedure may also lead to oropharyngeal injuries, although none occurred in our study. The second difficulty was the insertion of the tube between the vocal cords. Since the tube’s tip was also hyperangulated and positioned upwards by the stylet, it is hard to place the tube into the vocal cords. Moreover, anesthesia specialists generally have greater clinical experience with the Macintosh blade, even though they are also familiar with other airway devices. This might also potentially lengthen intubation time when using the D-Blade. Therefore, we think the MAC blade could be a better option for inexperienced practitioners in pediatric patients with difficult airways.

Two patients were excluded following failed intubation in the D-Blade group due to the methodology of the study. Since Fiadjoe et al. [1] showed that airway complications were associated with multiple tracheal intubation attempts (>2), our study was designed around a single attempt at intubation due to ethical concerns. Kleine-Brueggeney et al. [8] reported that first-attempt success rates were highest using devices with blades that were small enough to be introduced and adjusted inside the oral cavity. That study also showed that intubation failures were often associated with tube advancement. The authors recommended that stylets should be used when using indirect VLs without a guiding channel and suggested that TI may still be challenging even with an optimally shaped stylet.

This study has several limitations. The first is the possibility of bias since the anesthetists could not be blinded to the study groups. The patients in our study were also healthy individuals, and the DA scenario was simulated using neck collars. This scenario is very different from daily clinical practice, in which the airway may be edematous, obstructed, or not clear because of secretions. Therefore, the results in different scenarios might differ from our findings, and we cannot know which VL would be best for each situation. We think that in addition to clinical availability, clinicians should decide between the blades according to their own experience and the type of airway scenario involved. Another limitation is that we only compared two blades from the same device, although many other options are becoming available. Additional prospective randomized studies are needed to compare different devices and the same type of blades between devices to determine both blade types and devices’ relative efficacies.

In conclusion, this study compared the Storz® C-Mac VL Macintosh blade with D-Blade in a simulated pediatric DA scenario. The results showed that a better laryngoscopic view was obtained with D-Blade. However, the Storz® C-Mac videolaryngoscope Macintosh blade was superior to the D-Blade in achieving a shorter time for TI.

## Figures and Tables

**Table 1:** Demographic data.

	Mac group (n = 21)	D-Blade group (n = 20)	P
Age (year)	2.5 ± 1.3	2.5 ± 1.2	0.872
Weight (kg)	13.5 ± 4.7	13 ± 3.8	0.831
Height (cm)	91.2 ± 6.4	92.4 ± 5.9	0.769
ASA status (I/II)	11/10	14/8	0.466

Data are presented as mean ± SD and patient numbers.

**Table 2:** Comparison of MCLS scores.

	MCLS 1	MCLS 2A	p
MAC group	9	12	**0.03**
D-Blade group	15	5	
